# Synthesis and Evaluation of Novel α-Aminoamides Containing an Indole Moiety for the Treatment of Neuropathic Pain

**DOI:** 10.3390/molecules21070793

**Published:** 2016-06-23

**Authors:** Haotian Li, Shiyong Fan, Jingchao Cheng, Ping Zhang, Bohua Zhong, Weiguo Shi

**Affiliations:** Beijing Institute of Pharmacology & Toxicology, 27 Tai-Ping Road, Beijing 100850, China; swordsman_0526@163.com (H.L.); fsyn1996@163.com (S.F.); chengjingchao3017@126.com (J.C.); gundnir@163.com (P.Z.)

**Keywords:** neuropathic pain, α-aminoamide derivatives, voltage-gated sodium channel blockers

## Abstract

The α-aminoamide family of sodium ion channel blockers have exhibited analgesic effects on neuropathic pain. Here, a series of novel α-aminoamides containing an indole ring were designed and synthesized. These compounds were evaluated in mice using a formalin test and they exhibited significant anti-allodynia activities. However, the analgesic mechanism of these compounds remains unclear; a subset of the synthesized compounds can only moderately inhibit the sodium ion channel, Nav1.7, in a whole-cell patch clamp assay. Overall, these results suggest that introduction of an indole moiety to α-aminoamide derivatives can significantly improve their bioactivity and further study is warranted.

## 1. Introduction

Neuropathic pain is a chronic disease caused by lesion or dysfunction of the somatosensory system [1,2]. As a result, neuropathic pain reduces the quality of life of afflicted patients and increases the cost of their medical care. Currently, there is a lack of specific analgesics to treat this clinical problem [3,4].

**Figure 1:**
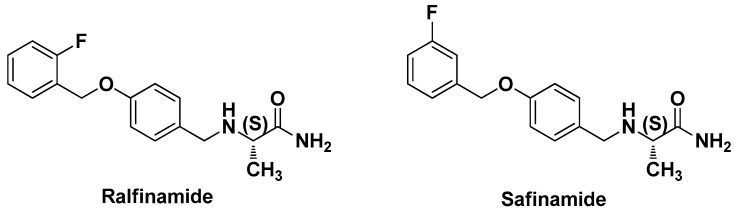
Chemical structures of ralfinamide and safinamide.

Recent studies have suggested that voltage-gated sodium channels (VGSCs) may be an ideal therapeutic target for neuropathic pain. Some α-aminoamide derivatives, including ralfinamide (Figure 1), are potential anti-neuropathic drug candidates. Ralfinamide is an orally active selective blocker of the VGSC, Nav1.7, and has been developed by Newron Pharmaceuticals [5]. Data from animal models of inflammatory and neuropathic pain have suggested that ralfinamide exhibits a significant analgesic effect while also having a well-tolerated safety profile [6,7,8]. Moreover, ralfinamide is the only small molecule drug in a late-stage clinical trial for the treatment of neuropathic pain [9,10,11,12,13]. However, the anti-allodynic efficacy and Nav1.7-selectivity of ralfinamide need further improvement.

Safinamide (Figure 1), another α-aminoamide derivative, has been approved by the European Commission in February 2015 for the treatment of Parkinson’s disease. In contrast with the activity of ralfinamide, safinamide exhibits selective and reversible monoamine oxidase B (MAO-B) inhibitory activity [14,15,16]. The only structural difference between ralfinamide and safinamide is the position of the fluoro substituent (Figure 1). Such slight chemical structural differences between ralfinamide and safinamide result in dramatic changes in pharmacological activity, indicating that delicate structure-activity relationship (SAR) inspection is needed for these types of compounds.

**Figure 2:**
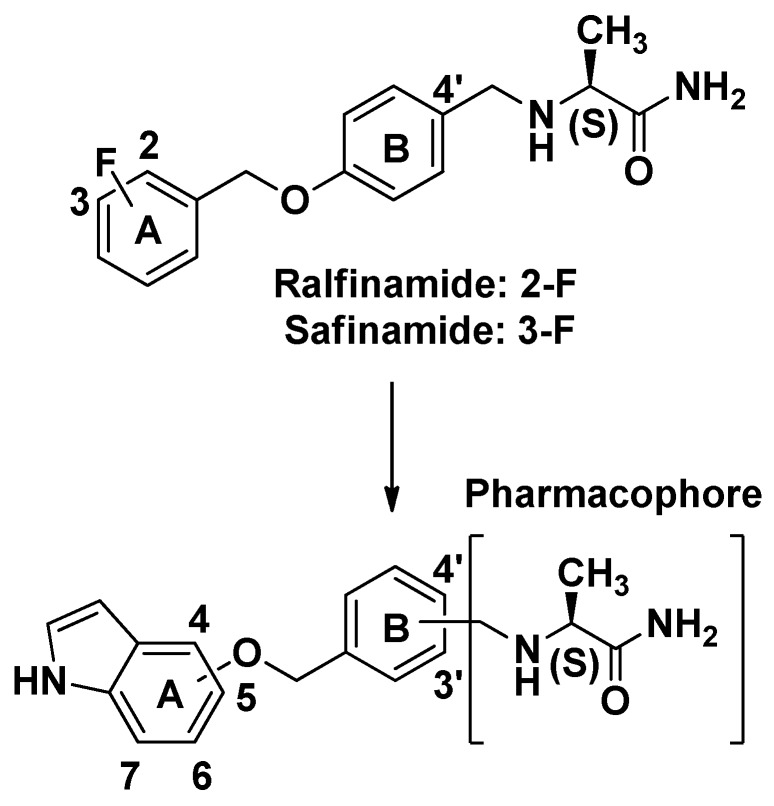
The design of novel α-aminoamide derivatives. Substitutions were made in the A ring at positions 4, 5, 6 or 7. Substitutions involving the α-aminoamide pharmacophore were made in the B ring at either the 3’ or 4’ position.

Here, a set of novel compounds that contain an indole ring and the α-aminoamide pharmacophore were designed (Figure 2). According to recent studies, indole derivatives can also exhibit Na^+^ channel inhibitory activity [17]. Therefore, we replaced the A ring of ralfinamide with an indole ring and varied the substitution position of the indole ring and the pharmacophore (Figure 2). Meanwhile, the position of the oxygen atom between the A ring and B ring was different to that of ralfinamide, which plays a critical role in the inhibitory activity and specific target selectivity based on our previous study. The bioactivity of the resulting compounds for treating neuropathic pain were subsequently evaluated and compared in this paper.

## 2. Results and Discussion

### 2.1. Synthesis

**Scheme 1:**
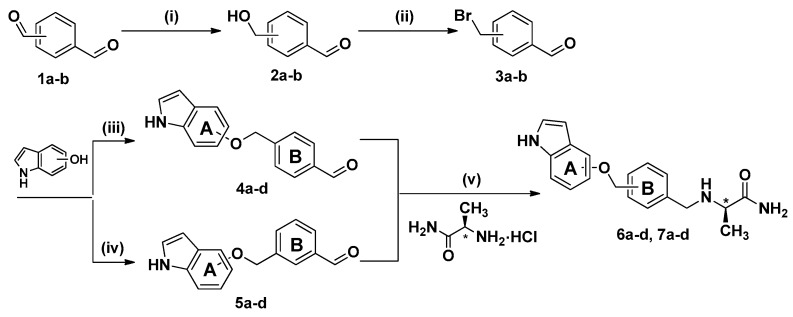
Synthesis of compounds **6a**–**d** and **7a**–**d**. *Reagents and Conditions*: (i) NaBH_4_, C_2_H_5_OH, THF, ice-bath, 6 h; (ii) NBS, PPh_3_, DCM, 4.5 h; (iii) K_2_CO_3_, KI, acetone, 58 °C, 24 h; (iv) Cs_2_CO_3_, DMF, 40 °C, overnight; (v) l-alaninamide hydrochloride, NaBH_3_CN, MeOH, 8 h.

As shown in Scheme 1, target compounds **6a**–**d** and **7a**–**d** were synthesized in four steps. First, the aldehyde group of terephthalaldehyde (**1a**) or isophthalaldehyde (**1b**) was reduced by sodium borohydride, then brominated with NBS using PPh_3_ as a catalyst to obtain the intermediate **3a** and **3b**, respectively. Next, **3a** and **3b** were etherified with hydroxyindoles by Williamson ether synthesis. Finally, **4a**–**d** and **5a**–**d** were converted into a series of α-aminoamide derivatives by reductive amination.

### 2.2. Analgesic Activity of Target Compounds in Formalin Test

We firstly screened the *in vivo* activities of the compounds in mice using a formalin test, because our studies have suggested this type of compounds with the oxygen atom at this position exhibited lower in vitro Nav1.7 inhibitory activity than that of ralfinamide, so the inhibitory activity against Nav1.7 was not sufficient for showing their real analgesic efficiency. We tested the effect of the compounds at a single dosage of 10 mg/kg, according to the effective doses resulting in a 50% reduction (ED_50_; 7.3 (5.16–11.28) mg/kg) of ralfinamide; and further evaluated the minimum effective dosage of the most potent compound, **6a**, compared to that of ralfinamide. 

As shown in Table 1, all tested compounds exhibited significant analgesic efficacy in phase 2 of the formalin test (*p*-value < 0.05; compared with the vehicle group). The SAR studies have suggested that analgesia of target compounds with substitution in the B ring at 4’ position seems slightly better than that of compounds at 3’ position, except for **6b** and **7b**. In particular, intraperitoneal (i.p.) administration of compound **6a** at a dose of 10 mg/kg resulted in 76.3% analgesia potency.

**Figure 3:**
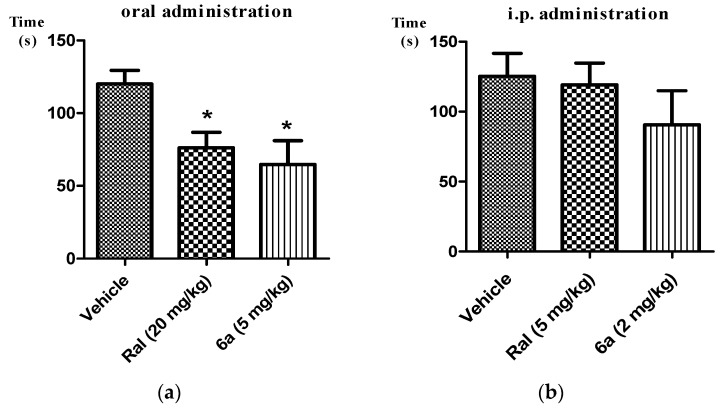
Cumulative duration of licking time of injected paw following oral (**a**) and i.p. (**b**) administration of ralfinamide (ral) and compound **6a** during the formalin test. Significant differences between vehicle and drug-treated groups were evaluated by the Dunnett’s test. * *p* < 0.05.

Further investigation demonstrated that compound **6a** exhibited 46.1% and 27.8% analgesia upon oral administration at 5 mg/kg and i.p. administration at 2 mg/kg, respectively. These effects were more potent than that observed for ralfinamide, which exhibited only 36.5% and 4.9% analgesia upon oral administration at 20 mg/kg and i.p. administration at 5 mg/kg, respectively. The raw data for cumulative licking time are presented in Figure 3.

### 2.3. Inhibitory Activities of the Tested Compounds on the Tetrodoxin-Sensitive and Inactivated Human-Nav1.7 (hNav1.7)

Tested compounds **6a**, **7a**, and **7b** exhibited good analgesic activity in the formalin test and then were evaluated for in vitro activity with whole cell patch-clamping assays of recombinant HEK293 cells expressing Nav1.7. The inhibitory activities of the tested compounds are listed in Table 2. The compounds were screened at a single dose of 10 μM according to the IC_50_ of ralfinamide (7.10 ± 1.41 μM). As shown in Table 2, In agreement with our anticipation, the tested compound **6a** can hardly inhibit hNav1.7, whereas tested compounds **7a** and **7b** can moderately inhibit hNav1.7. Data have indicated that substituted position of methylene in the B ring may result in variation of Nav1.7-selective inhibitory activity.

Neuropathic pain is one of the most complex pain syndromes to manage, resulting in a major clinical and socioeconomic problem. The therapeutic analgesics currently available are nonspecific and ineffective in many cases of neuropathic pain. Studies have suggested that ralfinamide, a well-tolerated small molecule α-aminoamide derivative targeting Nav1.7, was once developed as the most promising drug candidate for the specific treatment of neuropathic pain. Unfortunately, ralfinamide failed in a phase IIb/III clinical trial for treating neuropathic lower back pain; and the channel subtype selectivity and analgesic potency of ralfinamide need to be further improved. Thus, we designed and synthesized a set of novel α-aminoamide derivatives based on the modified chemical structure of ralfinamide by introduceing indole moiety to replace the substituted benzene ring in ralfinamide and evaluated their biological activities.

We first tested the analgesic activities of the compounds in formalin mice model. In very good agreement with our design, novel compounds containing the indole group exhibited greater in vivo potency compared with that of ralfinamide. Thus we further tested the in vitro inhibitory activities of these compounds on Nav1.7. Surprisingly, they displayed lower Nav1.7 selectivity than that of ralfinamide, indicating that Nav1.7 might not be the real target for these new compounds and the mechanism of action of these compounds needed to be further investigated. For SAR investigation, no reliable relations have been found for the substitution in the indole ring according the results of formalin test; although the substitution in the position 7 seems to be less active that others. The inhibition of the Nav1.7 channel is higher in the meta derivatives (B ring), which opposes to the results found in the formalin assay, indicating that the meta position was crucial for the channel selectivity of the new compounds. Additional assays of these compounds as inhibitors for Nav1.8 and Nav1.9 as well as other ion channels concerned with pain should be performed and disclosed in due course.

## 3. Experimental Section

### 3.1. General Information

Reagents were obtained from Beijing Chemical Works (Beijing, China), Acros Organics (Geel, Belgium) and Alfa-Aesar (Ward Hill, MA, USA), and were used without further purification. Proton nuclear magnetic resonance (^1^H-NMR, 400 MHz) spectra were measured with a JNM-ECA-400 spectrometer (JEOL Co. Ltd., Tokyo, Japan). Mass spectra were measured by using an API-150 mass spectrometer (ABI Inc., Foster City, CA, USA) with an 1100-HPLC electrospray ionization source (Agilent Technologies Inc., Palo Alto, CA, USA).

### 3.2. Chemistry

#### 3.2.1. Synthesis of 4-(Hydroxymethyl)benzaldehyde (**2a**) and 3-(Hydroxymethyl)benzaldehyde (**2b**)

NaBH_4_ (1.7 g) was added to a solution of terephthalaldehyde (**1a**, 20.0 g) or isophthalaldehyde (**1b**, 20.0 g) in ethanol (100 mL) and tetrahydrofuran (150 mL). The reaction was stirred in an ice-bath for 6 h. After reaction completion, the solution was quenched with 2 M hydrochloric acid to the pH 5–6. The solvent was evaporated, then water and ethyl acetate were added to the residue. The organic phase was washed with a saturated NaCl and dried with Na_2_SO_4_ for 8 h. The mixture was purified by silica gel chromatography with petroleum ether-ethyl acetate = 5:1 as eluent to give compound **2a** 17.6 g (86.1% yield) and **2b** 16.4 g (80.2% yield), respectively. 

#### 3.2.2. Synthesis of 4-(Bromomethyl)benzaldehyde (**3a**) and 3-(Bromomethyl)benzaldehyde (**3b**)

*N*-Bromosuccinimide (NBS, 19.6 g) was added to a solution of **2a** or **2b** (10.0 g) in dichloromethane (120 mL). Triphenylphosphine (2.0 equiv, 38.5 g, 0.146 mol) was divided into four equal aliquots and an aliquot was added to the reaction every 30 min. After the reaction was completed, the solution was mixed with cold water (120 mL). The aqueous phase was extracted two times with dichloromethane and the organic phase was washed with a saturated NaCl solution and dried over Na_2_SO_4_ for 8 h. The mixture was purified by silica gel chromatography with petroleum ether–ethyl acetate = 25:1 as eluent to give compound **3a** 8.5 g (58.0% yield) and **3b** 6.8 g (46.8% yield), respectively.

#### 3.2.3. Preparation of Intermediates **4a**–**d**

Potassium carbonate (3.0 equiv.), potassium iodide (0.6 equiv.), and 4-, 5-, 6-, or 7-hydroxyindole (1.1 equiv.) were added to a solution of **3a** (1.0 equiv.) in acetone (100 mL). The mixture was stirred and heated to 58 °C for 24 h. The solution was filtered and the solvent was evaporated. Ethyl acetate was added to the residue, the organic phase was washed with 0.5 N NaOH and a saturated NaCl solution. Products were purified by silica gel column chromatography using petroleum ether:ethyl acetate = 5:1 as the eluent to give the compounds **4a**–**d**.

#### 3.2.4. Preparation of Intermediates **5a**–**d**

Cesium carbonate (1.5 equiv.), potassium iodide (0.6 equiv.), and 4-, 5-, 6-, or 7-hydroxyindole (1.1 equiv.) were added to a solution of **3b** (1.0 equiv.) in dimethylformamide (DMF, 40 mL). The mixture was stirred and heated to 40 °C for 12 h. The solution was filtered and the solvent was evaporated. Following the addition of ethyl acetate to the residue, the organic phase was washed with 0.5 N NaOH and a saturated NaCl solution. The products were purified with silica gel column chromatography using petroleum ether-ethyl acetate = 5:1 as the eluent to give the compounds **5a**–**d**.

#### 3.2.5. Preparation of Target Compounds **6a**–**d**

A solution of L-alaninamide hydrochloride (1.1 equiv.), sodium cyanoborohydride (0.8 equiv.), 3Å molecular sieve (1 g), and triethylamine (1 mL) in methanol (80 mL) was stirred at room temperature for 15 min. Then, **4a**–**d** (1.0 equiv.) were added rapidly to the reaction mixture. The mixture was stirred at 40 °C for 8 h. After reaction completion, the solution was filtered and the solvent was evaporated. Water (300 mL) and ethyl acetate (500 mL) were added to the residue, the organic phase was washed with a saturated NaCl solution and dried over Na_2_SO_4_ for 8 h. The products were purified by silica gel column chromatography using methanol–dichloromethane = 5:1 as the eluent to give the compounds **6a**–**d** as grey solids in 33.3%–41.0% yield. 

*(S)-2-((4-(((1H-Indol-4-yl)oxy)methyl)benzyl)amino)propanamide* (**6a**): ^1^H-NMR δ_H_ (DMSO-*d*_6_, ppm): 10.92 (s, 1H, NH indole), 6.98–7.43 (m, 11H, H_Ar_ and NH_2_), 5.06 (s, 2H, O-CH_2_), 3.54–3.71 (m, 2H, C**H**_2_-NH), 2.99–3.02 (m, 1H, C**H**-CH_3_), 1.13 (d, 3H, *J* = 6.7 Hz, CH_3_). MS (ESI) *m*/*z*: 323.2 (M + H).

*(S)-2-((4-(((1H-Indol-5-yl)oxy)methyl)benzyl)amino)propanamide* (**6b**): ^1^H-NMR δ_H_ (DMSO-*d*_6_, ppm): 10.85 (s, 1H, NH indole), 6.98–7.43 (m, 11H, H_Ar_ and NH_2_), 5.06 (s, 2H, O-CH_2_), 3.53–3.70 (m, 2H, C**H**_2_-NH), 2.99–3.02 (m, 1H, C**H**-CH_3_), 1.13 (d, 3H, *J* = 6.7 Hz, CH_3_). MS (ESI) *m*/*z*: 323.2 (M + H).

*(S)-2-((4-(((1H-Indol-6-yl)oxy)methyl)benzyl)amino)propanamide* (**6c**): ^1^H-NMR δ_H_ (DMSO-*d*_6_, ppm): 10.68 (s, 1H, NH indole), 6.98–7.40 (m, 11H, H_Ar_ and NH_2_), 5.04 (s, 2H, O-CH_2_), 3.53–3.70 (m, 2H, C**H**_2_-NH), 2.99–3.02 (m, 1H, C**H**-CH_3_), 1.12 (d, 3H, *J* = 6.7 Hz, CH_3_). MS (ESI) *m*/*z*: 323.2 (M + H).

*(S)-2-((4-(((1H-Indol-7-yl)oxy)methyl)benzyl)amino)propanamide* (**6d**): ^1^H-NMR δ_H_ (DMSO-*d*_6_, ppm): 10.74 (s, 1H, NH indole), 6.98–7.40 (m, 11H, H_Ar_ and NH_2_), 5.04 (s, 2H, O-CH_2_), 3.53–3.70 (m, 2H, C**H**_2_-NH), 3.09–3.13 (m, 1H, C**H**-CH_3_), 1.14 (d, 3H, *J* = 6.7 Hz, CH_3_). MS (ESI) *m*/*z*: 323.2 (M + H). 

The hydrogen atoms of the aliphatic NH groups, which are highly split due to the surrounding groups, are not visible in the NMR of these compounds.

#### 3.2.6. Preparation of Target Compounds **7a**–**d**

Compounds **7a**–**d** were synthesized by a similar procedure as **6a**–**d**, except compounds **4a**–**d** were replaced by **5a**–**d** at this reaction procedure. The resulting compounds **7a**–**d** were obtained as grey solids in yields of 31.0%–43.5%.

*(S)-2-((3-(((1H-Indol-4-yl)oxy)methyl)benzyl)amino)propanamide* (**7a**): ^1^H-NMR δ_H_ (DMSO-*d*_6_, ppm): 10.75 (s, 1H, NH indole), 6.99–7.40 (m, 11H, H_Ar_ and NH_2_), 5.05 (s, 2H, O-CH_2_), 3.55–3.71 (m, 2H, C**H**_2_-NH), 2.99–3.02 (m, 1H, C**H**-CH_3_), 1.14 (d, 3H, *J* = 6.7 Hz, CH_3_). MS (ESI) *m*/*z*: 323.2 (M + H).

*(S)-2-((3-(((1H-Indol-5-yl)oxy)methyl)benzyl)amino)propanamide* (**7b**): ^1^H-NMR δ_H_ (DMSO-*d*_6_, ppm): 10.77 (s, 1H, NH indole), 6.99–7.40 (m, 11H, H_Ar_ and NH_2_), 5.05 (s, 2H, O-CH_2_), 3.55–3.73 (m, 2H, C**H**_2_-NH), 3.01–3.05 (m, 1H, C**H**-CH_3_), 1.12 (d, 3H, *J* = 6.7 Hz, CH_3_). MS (ESI) *m*/*z*: 323.2 (M + H).

*(S)-2-((3-(((1H-Indol-6-yl)oxy)methyl)benzyl)amino)propanamide* (**7c**): ^1^H-NMR δ_H_ (DMSO-*d*_6_, ppm): 10.82 (s, 1H, NH indole), 6.99–7.45 (m, 11H, H_Ar_ and NH_2_), 5.08 (s, 2H, O-CH_2_), 3.55–3.73 (m, 2H, C**H**_2_-NH), 3.02–3.05 (m, 1H, C**H**-CH_3_), 1.14 (d, 3H, *J* = 6.7 Hz, CH_3_). MS (ESI) *m*/*z*: 323.2 (M + H).

*(S)-2-((3-(((1H-Indol-7-yl)oxy)methyl)benzyl)amino)propanamide* (**7d**): ^1^H-NMR δ_H_ (DMSO-*d*_6_, ppm): 10.89 (s, 1H, NH indole), 7.02–7.51 (m, 11H, H_Ar_ and NH_2_), 5.12 (s, 2H, O-CH_2_), 3.65–3.78 (m, 2H, C**H**_2_-NH), 3.05–3.08 (m, 1H, C**H**-CH_3_), 1.17 (d, 3H, *J* = 6.7 Hz, CH_3_). MS (ESI) *m*/*z*: 323.2 (M + H).

The hydrogen atoms of the aliphatic NH groups, which are highly split due to the surrounding groups, are not visible in the NMR of these compounds.

### 3.3. Formalin Test

The *in vivo* analgesic activities of the compounds in an animal model of neuropathic pain were evaluated using a formalin test in mice as previously described [6]. Briefly, the test compounds were dissolved in distilled water. ICR mice (weight: 22–25 g) were administered ralfinamide or one of the tested compounds by intraperitoneal route at a dose of 10 mg/kg in a volume of 10 mL/kg body weight (6 mice per group). Thirty min later, the mice were injected subcutaneously with 20 μL of 2.7% formalin on the plantar surface of the left hind paw and were placed into PVC observation chambers (23 × 12 × 13 cm^3^). Pain behavior during phase 2 was quantified by counting the time that each mouse licked the injected paw.

Data are presented as the mean ± standard error of the mean (SEM) of six animals per dose group and were evaluated by one-way analysis of variance (ANOVA) followed by Dunnett’s test. The raw data of cumulative licking time were converted to percent (%) analgesia according to the following formula:

$$\%\text{Analgesia Percentage }=\;100\times \frac{\text{Average Time }\left(\text{Vehicle}\right)-\text{Average Time }\left(\text{Test Drug}\right)}{\text{Average Time }\left(\text{Vehicle}\right)}$$

### 3.4. Electrophysiology

Whole-cell voltage clamp recordings were performed at room temperature on recombinant HEK293 cells that expressed the human VGSC subtype 1.7 (Nav1.7) using an EPC 10 amplifier (HEKA Electronics, Pfalz, Germany). To measure inactivated Nav1.7 channels, the currents were recorded under a holding potential at −120 mV and then were depolarized to 0 mV for 1 s to inactivate the sodium currents (TP1). Then the membrane potential was repolarized to −120 mV for 20 ms, followed by a depolarized pulse to 0 mV (TP2). The peak currents of TP1 and TP2 were recorded and were analyzed independently. An interpulse interval of 10 s was employed to allow recovery from inactivation. Fast perfusion was used to directly apply the drugs tested to the cells in seconds. Each concentration was perfused over 2 min, or until the current reached a steady-state level. After the final concentration of the reference agent was tested, the reference compound was washed out with extracellular solution for 5 min. Within each recording, current responses to the addition of tested compounds were normalized to the vehicle control (=I_compound_/I_vehicle control_) and the ratios of inhibition (=1 − current response/maximal control tail current × 100%) were calculated. Mean values and standard errors were calculated for each test group.

## 4. Conclusions

In conclusion, novel highly potent α-aminoamide derivatives for the treatment of neuropathic pain containing indole moieties were designed and synthesized. We found that these novel compounds exhibited significant inhibitory activity in the *in vivo* formalin-induced pain model test, superior to that of the positive control ralfinamide. However, the novel compounds displayed lower Nav1.7 selectivity compared with that of ralfinamide, indicating different mechanisms of action between ralfinamide and the designed samples. Further investigations need to be performed and will be disclosed in due course. 

## Figures and Tables

**Table 1:**
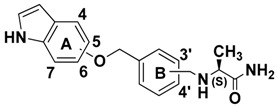
*In vivo* activity of the synthesized compounds in the formalin test performed.

Compounds	Substituted Position of the Oxygen Atom in the A Ring	Substituted Position of –CH_2_– in the B Ring	% Analgesia ^a^ (10 mg/kg, i.p.)
**6a**	4	4’	76.3 ± 3.9
**6b**	5	4’	62.1 ± 2.8
**6c**	6	4’	71.0 ± 1.0
**6d**	7	4’	68.6 ± 1.8
**7a**	4	3’	64.9 ± 4.7
**7b**	5	3’	67.2 ± 6.3
**7c**	6	3’	68.8 ± 4.9
**7d**	7	3’	58.5 ± 5.9
**Ralfinamide**	-	-	47.5 ± 2.9

^a^ Data from the formalin tests performed are presented as the mean ± standard deviation.

**Table 2:**
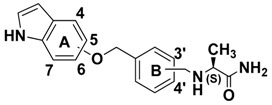
Inhibitory activities of select compounds on the tetrodotoxin-sensitive and inactivated Nav1.7.

Compound	Substituted Position of the Oxygen Atom in the A Ring	Substituted Position of –CH_2_– in the B Ring	hNav1.7 (% Inhibition at 10 μM)
**6a**	4	4’	4 ± 2
**7a**	4	3’	59 ± 7
**7b**	5	3’	29 ± 2
**Ralfinamide**	-	-	66 ± 5
